# A Prospective, Randomized, Active-Controlled, Evaluator-Blinded, Noninferiority Clinical Trial Examining a Non-Cross-Linked Bovine-Derived Type I/III Collagen Filler Combined with Hyaluronic Acid for Periocular Rejuvenation

**DOI:** 10.1007/s00266-025-05499-z

**Published:** 2025-12-04

**Authors:** Guo-Sheng Zhu, Si-Yi Zhang, Lu-Wen Deng, Fang Yang, Xiang Wang, Hong-Yan Wang, Hong-Qiang Li, Wei-Jin Hong, Sheng-Kang Luo

**Affiliations:** 1 Department of Plastic and Reconstructive Surgery, the Affiliated Guangdong Second Provincial General Hospital of Jinan University, 466 Middle Xin Gang Road, Guangzhou City, 510317 Guangdong Province People’s Republic of China; 2 Shenzhen Yixing Medical Aesthetic Hospital, 3024 Shen Nan Zhong Road, Huaqiangbei Street, Hua Hang Community, Futian District, Shenzhen City, 518000 Guangdong Province People’s Republic of China; 3 Huahan Group Sichuan Yuehao Medical Aesthetic Hospital, 335 Shuhan Road, Jinniu District, Chengdu City, 610000 Sichuan Province People’s Republic of China; 4 COCOAEST Medical Aesthetic Clinic, 6 Shandong Road, Shinan District, Qingdao City, 266555 Shandong Province People’s Republic of China; 5 Feiman (Changchun) Pharmaceutical Biotechnology Co., Ltd., 6645 Xincheng Street, Jingyue Development Zone, Changchun City, 130000 Jilin Province People’s Republic of China

**Keywords:** Collagen, Hyaluronic acid, Tear trough deformity, Periocular aging, Injection technique

## Abstract

**Background:**

The use of dermal fillers for periocular rejuvenation is common, with hyaluronic acid (HA) being the most popular agent. However, the use of HA in the delicate tear trough region may induce the Tyndall effect, resulting in undesirable bluish discoloration of the lower eyelid. The main drawback of collagen monotherapy is its relatively short effective duration. In this study, FILLDERM^TM^ (manufactured by Jilin Changchun Botai Pharmaceutical Co., Ltd., China) and Restylane^®^ (produced by Galderma SA, Switzerland) were used as the primary treatments.

**Objectives:**

This study evaluated the efficacy and safety of combining collagen with HA for moderate periocular aging. This combination approach was compared with HA and collagen monotherapies.

**Methods:**

Patients were randomized to receive (1) HA alone, (2) collagen alone, or (3) a combination of HA and collagen via our standardized dual-plane injection protocol (3 sharp-needle periosteal injections + 1 cannula subdermal injection). The treatment outcomes were assessed using the Allergan Infraorbital Hollow Scale (AIHS), the Global Aesthetic Improvement Scale (GAIS), and standardized photographic evaluation at multiple follow-ups.

**Results:**

Compared with monotherapies, the combination therapy yielded superior outcomes in terms of periocular volume restoration and longevity. The combination therapy effectively addressed both structural support (via periosteal injections) and superficial rejuvenation (via subdermal placement) while significantly reducing the incidence of the Tyndall effect in the HA monotherapy group. The combination therapy showed extended durability compared with collagen monotherapy.

**Conclusions:**

The combination of collagen and HA offers a comprehensive approach for periocular rejuvenation. This method combines the volumizing benefits of HA with the tissue-regenerative properties of collagen. Furthermore, this approach minimizes complications while enhancing treatment longevity, thus establishing a new standard for minimally invasive periocular rejuvenation.

**Level of Evidence I:**

This journal requires that authors assign a level of evidence to each article. For a full description of these Evidence-Based Medicine ratings, please refer to the Table of Contents or the online Instructions to Authors www.springer.com/00266.

**Supplementary Information:**

The online version contains supplementary material available at 10.1007/s00266-025-05499-z.

## Introduction

The periorbital region is one of the most anatomically delicate and aesthetically significant areas of the face; it serves as a primary focal point during human interaction and emotional expression [1]. Tear trough deformity is a key indicator of facial aging and presents as a distinct concave depression extending from the medial canthus to the mid-pupillary line, casting characteristic shadows that convey fatigue and aging [2].

The pathogenesis of tear trough deformity involves complex anatomical changes across multiple tissue layers [3] At a superficial level, the weakening of the orbicularis oculi muscle and the thinning of the dermal collagen network contribute to skin laxity. At a structural level, the weakening of the orbitomalar ligament combined with the pseudoherniation of orbital fat leads to the characteristic double-convexity deformity [4]. Furthermore, the age-induced resorption of the maxillary and orbital bones exacerbates the hollowing effect, while postinflammatory hyperpigmentation often accentuates the visual prominence of the deformity.

Clinical observations have shown that tear trough deformities are no longer restricted to older individuals but are now increasingly appearing in younger populations, often as early as the third decade of life [5]. While traditional surgical approaches, such as blepharoplasty and fat repositioning, are effective to treating severe cases, they carry the risks of overcorrection, prolonged recovery, and unnatural aesthetic outcomes. These limitations have made injectable fillers the treatment of choice for nonsurgical tear trough rejuvenation [6, 7].

Hyaluronic acid (HA) fillers are commonly used due to their biocompatibility, reversibility, and integration with surrounding tissue [7, 8]. However, the application of HA in the thin, delicate skin of the periorbital region presents challenges. The Tyndall effect—a bluish discoloration that can occur due to superficial injection of HA—remains a significant complication, with incidence rates ranging from 15 to 30%. Moreover, the dynamic nature of the periorbital area accelerates HA degradation; therefore, treatments need to be repeated every 6–9 months to maintain results.

Collagen-based fillers offer distinct advantages, including superior light-scattering properties that help prevent the Tyndall effect. Additionally, collagen is naturally compatible with dermal tissue architecture. However, collagen fillers are generally limited by their shorter effective duration—typically ranging from 3–4 months—and variable tissue integration [9, 10]

To address these complementary limitations, we developed an optimized combination approach in which collagen filler is directly mixed with low-molecular-weight HA. In this study, the collagen-based filler used was FILLDERM^TM^ (manufactured by Jilin Changchun Botai Pharmaceutical Co., Ltd., China) and Restylane® (produced by Galderma SA, Switzerland) was used for comparison. Both products have a long-standing history of clinical use in facial aesthetics. FILLDERM^TM^ is derived from bovine sources and consists of Type I and Type III collagen, providing volumizing effects. Restylane® is a non-animal stabilized hyaluronic acid (NASHA) filler, commonly used for long-lasting volumizing effects. This direct mixture of the two materials combines collagen’s immediate volumizing effect with HA’s longer-lasting volume maintenance properties, thereby offering a solution that addresses both the aesthetic and longevity challenges of periorbital rejuvenation.

## Patients and Methods

### Study Design

This prospective, randomized, evaluator-blinded, noninferiority clinical trial evaluated the efficacy and safety of combining FILLDERM^TM^ (3.5% bovine-derived Type I/III collagen) and Restylane^®^ (20 mg/mL hyaluronic acid) for periocular rejuvenation. Group A (collagen only) received collagen injections, Group B (HA only) received HA injections, and Group C (combination) received a 1:1 ratio of collagen and HA. The maximum injection volume for each group was 2 mL per side. This study was approved by the Affiliated Guangdong Second Provincial General Hospital of Jinan University (2022-QXLCYJ-003). The trial ran from May 22, 2023, to February 22, 2024, and participants were randomly assigned to one of the three groups using a computer-generated randomization list.

**Fig. 1:**
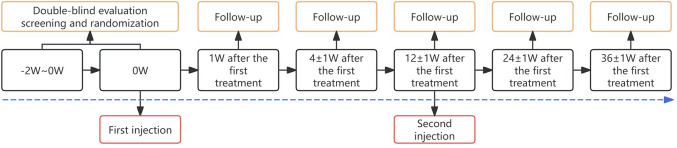
Flowchart of the treatment sessions and follow-up assessments

Participants underwent two treatment sessions: the initial treatment (T1) at baseline and a follow-up treatment (T2) 12 weeks later. Follow-up assessments were conducted at 1, 4, 12, 24 and 36 weeks after baseline (Fig. 1). During each follow-up assessment, standardized 3D photography (Canon EOS90D) was used to capture images in the front view, top view, upward view, and 45° lateral view. All assessments and adverse events were recorded in the case record form (CRF).

### Patients

A total of 45 subjects were screened and randomized into the three groups. Inclusion criteria included: (1) aged 18–65, (2) moderate-to-severe bilateral tear trough deformities (AIHS score 2-3), (3) ability to complete self-reported assessments, and (4) written informed consent. Exclusion criteria included: (1) active inflammatory skin conditions or facial surgeries within 6 months, (2) history of ocular surgery, (3) known allergies to study materials, (4) pregnancy or breastfeeding, or (5) significant medical conditions affecting outcomes.

### Injection Method

**Fig. 2:**
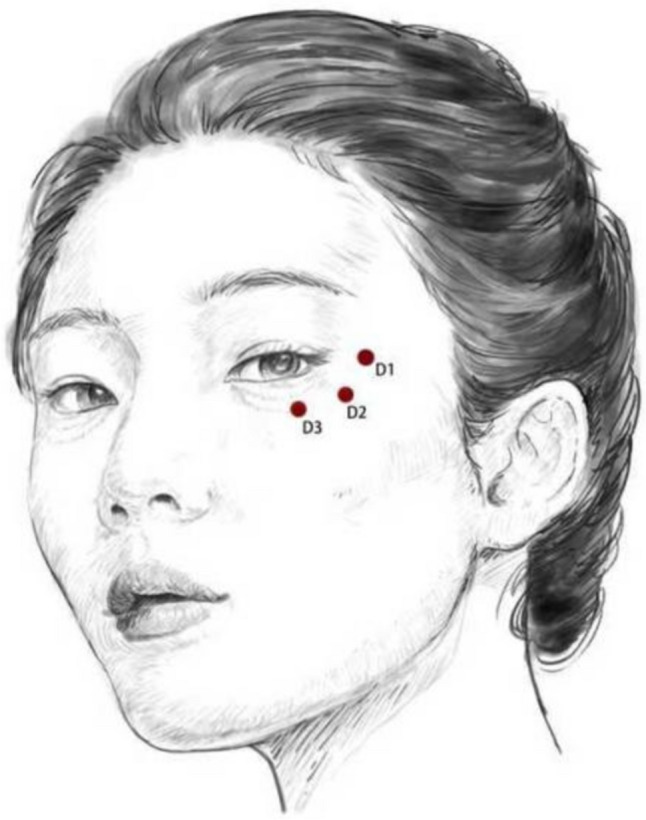
3 Needle injection sites for correcting tear troughs

**Fig. 3:**
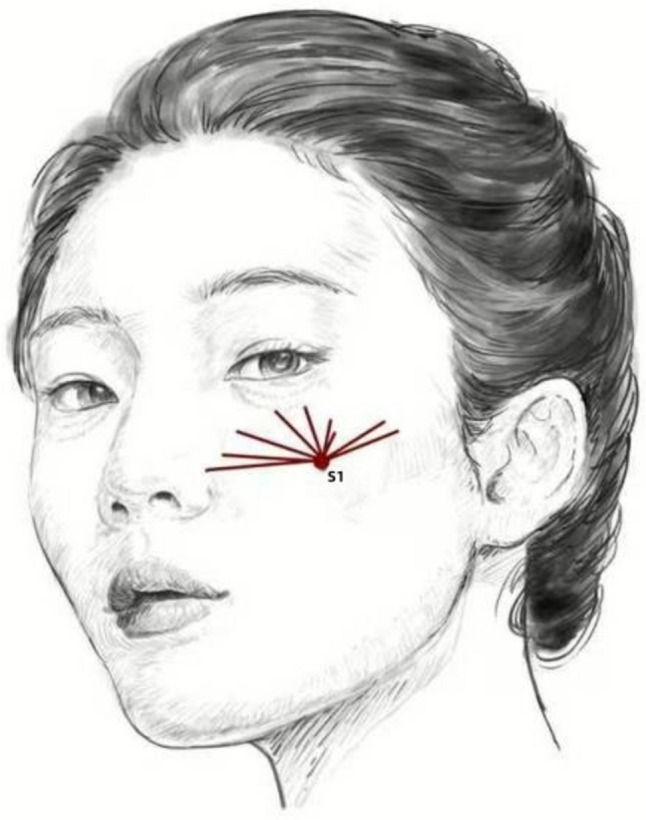
1 Cannula injection sites for correcting tear troughs

Injection interventions were conducted by a single experienced doctor. Patients were instructed to cleanse their facial skin and administer a 5% lidocaine cream as a topical surface anesthetic 40 minutes before the procedure. All three groups were subjected to the dual-plane injection protocol for the administration of collagen alone, HA alone, or the combination therapy. The technique combined needle-based periosteal injections (3 points) and cannula subdermal injections (1 point) to deliver a maximum volume of 2 mL per side. The needles (27G) were targeted at the following anatomical locations: D1 (lateral orbital retaining ligament, thickened lateral orbital region at the tail of the eyebrow), D2 (infraorbital retaining ligament, transition zone between the suborbicularis oculi fat (SOOF) and the medial cheek), and D3 (tear trough ligament, intersection of the tear trough, cheek, and midface) (Fig. 2). The cannula (25G Cannula) injections were focused on S1 (nasojugal zone, vertical line from the lateral canthus intersecting the horizontal line at the nasal alar notch) (Fig. 3, Video 1).

### Assessment and Safety Monitoring

AIHS and GAIS scores were recorded at baseline, postinjection, and at follow-up visits to assess periocular volume restoration and global aesthetic improvement. Adverse events, including edema, bruising, and Tyndall effect, were monitored at each follow-up. The severity and occurrence of complications were recorded, and appropriate interventions were made if needed.

### Statistical Analysis

Data were analyzed using IBM SPSS Statistics (version 26.0). Descriptive statistics were used to summarize baseline characteristics. For inter-group comparisons, the Kruskal–Wallis test was used for continuous variables, and chi-square tests were applied for categorical variables. Post hoc pairwise comparisons were performed using Mann–Whitney U tests, with Bonferroni correction applied to adjust for multiple comparisons. A *p* value of <0.05 was considered statistically significant.

## Results

This study aimed to include 45 patients who were evenly divided into three study groups. However, two subjects from Group B and one subject from Group C were lost to follow-up. Therefore, 42 subjects were ultimately included in the study, comprising 8 males (34.50 ± 5.47 years) and 34 females (37.62 ± 8.87 years). No significant differences in age or AHIS scores were found among the three groups at baseline. (Table 1)

**Table 1:** Patient demographics before the first injection

Characteristics	Value (%)
*Age at injection, year*	
Mean± SD	37.02 ± 8.37
Range	25-59
*Sex*	
Male	8(19.05%)
Female	34(80.95%)
*Group A*	
N	15
Age (mean ± SD)	38.53 ± 9.80
AIHS scale [median (P25, P75)]	2(2,2.25)
*Group B*	
N	13
Age (mean ± SD)	36.64 ± 8.54
AIHS scale [median (P25, P75)]	2(2,2.5)
*Group C*	
N	14
Age (mean ± SD)	35.69 ± 6.56
AIHS scale [median (P25, P75)]	2(2,2)

Improvement ratios were defined as the percentage of subjects with bilateral infraorbital improvement scores ≥ 1, divided by the total number of enrolled subjects and multiplied by 100%. For Group A (Collagen group), the improvement ratios were as follows: 100% at baseline, 100% at 1 week, 73.33% at 1 month, 46.67% at 3 months (pre-treatment), 100% at 3 months (post-treatment), 80% at 6 months, and 46.67% at 9 months. Group B (Hyaluronic Acid group) demonstrated consistent improvement with 100% at baseline, 100% at 1 week, 100% at 1 month, 92.31% at 3 months (pre-treatment), 100% at 3 months (post-treatment), 100% at 6 months, and 69.23% at 9 months. In Group C (Collagen + Hyaluronic Acid group), the improvement ratios were 100% at baseline, 100% at 1 week, 93.33% at 1 month, 78.57% at 3 months (pre-treatment), 100% at 3 months (post-treatment), 100% at 6 months, and 71.43% at 9 months.

**Fig. 4:**
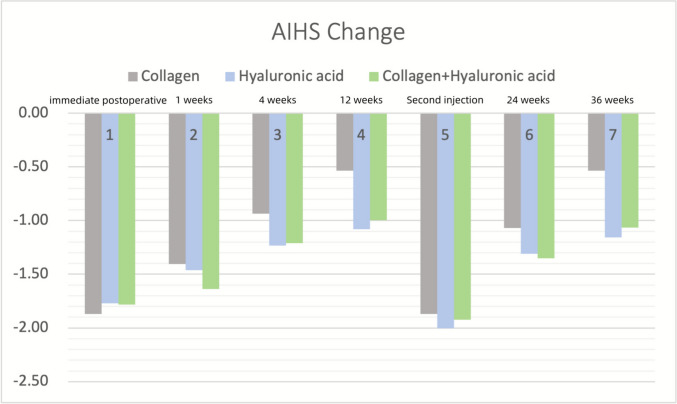
Changes in AIHS scores

When considering the AIHS improvement scores, a Kruskal–Wallis test was conducted to assess the statistical significance of the differences across the three groups (Group A: collagen injection, Group B: hyaluronic acid injection, and Group C: combination of collagen and hyaluronic acid) at each time point. At 1 week and 1 month, all groups demonstrated 100% improvement. However, at the 12-week follow-up, Group A showed a significant decline in improvement, with the improvement ratio dropping to 46.67%, the lowest observed at any time point. This suggests that while Group A showed initial improvement, its effects plateaued over time. Mann–Whitney U tests revealed significant differences between Group A (collagen) and Group B (hyaluronic acid) (*p* = 0.019), as well as between Group A (collagen) and Group C (collagen + hyaluronic acid) (*p* = 0.039), indicating that Group A's improvement was significantly lower than both Group B and Group C at 12 weeks. No significant difference was found between Group B (hyaluronic acid) and Group C (collagen + hyaluronic acid) (*p* = 0.752). At the 36-week follow-up, both Group B and Group C showed sustained improvement, while Group A's improvement plateaued. The Kruskal–Wallis test revealed significant differences between Group A and Group B (*p* = 0.035), as well as between Group A and Group C (*p* = 0.035). Mann–Whitney U tests further confirmed significant differences between Group A (collagen) and Group B (hyaluronic acid) (*p* = 0.012), and between Group A (collagen) and Group C (collagen + hyaluronic acid) (*p* = 0.006), while no significant difference was observed between Group B (hyaluronic acid) and Group C (collagen + hyaluronic acid) (*p* = 0.840). In summary, Group B (hyaluronic acid) and Group C (collagen + hyaluronic acid) demonstrated sustained and stable improvement over time, with no significant differences between these two groups. Group A (collagen) showed a plateau in improvement after the second injection, particularly at 3 months (pre-treatment), and had significantly lower improvement compared to Group B and Group C at both 12 weeks and 36 weeks (Fig. 4).

The safety profile of the treatments was evaluated by monitoring the occurrence of postinjection complications, including swelling, bruising, lumpiness, postinjection infection, and the Tyndall phenomenon. Group A (collagen injection) showed significantly lower rates of swelling compared to both Group B (hyaluronic acid) and Group C (collagen + hyaluronic acid), with a Chi-square test value of *p* = 0.03, indicating that Group A had a significantly lower incidence of swelling. No significant difference was found between any of the groups regarding bruising (*p* = 0.988) and lumpiness (*p* = 0.631), suggesting similar occurrences across all groups. No postinjection infections were reported in any group. Regarding the Tyndall phenomenon, no occurrence was observed in Group A (collagen) and Group C (collagen + hyaluronic acid), while Group B (hyaluronic acid) exhibited a significantly higher incidence, with a Chi-square test *p* value of 0.027. In summary, Group A (collagen) exhibited a lower incidence of swelling, while Group B (hyaluronic acid) showed a higher propensity for the Tyndall phenomenon. Group C (collagen + hyaluronic acid) demonstrated a well-balanced safety profile, with minimal complications and sustained improvement over time, offering a promising combination treatment option (Table 2).

**Table 2:** Postinjection complications

Complications	Group A	Group B	Group C
Swelling	6	12	12
Bruising	13	11	12
Lumpiness	3	4	5
Postinjection infection	0	0	0
Tyndall effect	0	3	0

**Fig. 5:**
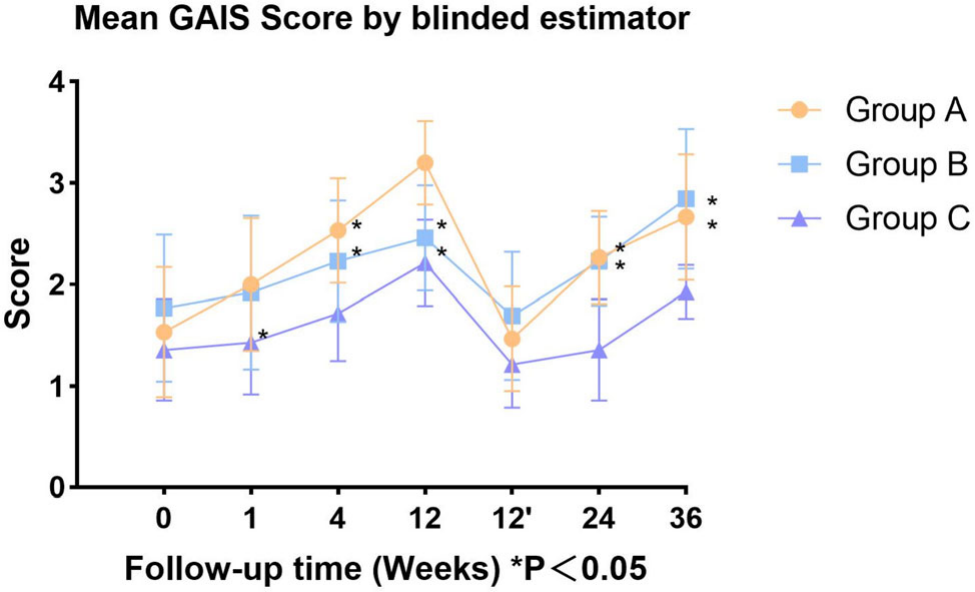
Mean investigator-rated GAIS scores

**Fig. 6:**
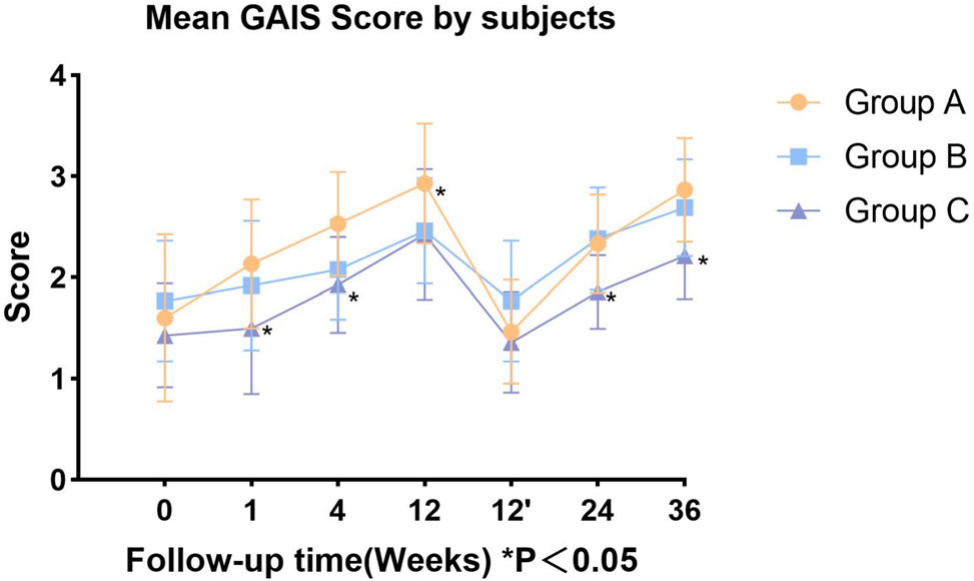
Mean self-reported GAIS scores

The mean investigator-rated GAIS scores in Group A (Fig. 5) were 1.53 ± 0.64, 2 ± 0.65, 2.53 ± 0.52, 3.2 ± 0.41, 1.47 ± 0.52, 2.27 ± 0.46, and 2.67 ± 0.62 at 0, 1, 4, 12, 24, and 36 weeks, respectively. The mean GAIS self-reported scores (Fig. 6) in Group A were 1.6 ± 0.83, 2.13 ± 0.64, 2.53 ± 0.52, 2.93 ± 0.59, 1.47 ± 0.52, 2.33 ± 0.49, and 2.87 ± 0.52 at 0, 1, 4, 12, 24, and 36 weeks, respectively. The mean investigator-rated GAIS scores in Group B were 1.77 ± 0.73, 1.92 ± 0.76, 2.23 ± 0.6, 2.46 ± 0.52, 1.69 ± 0.63, 2.23 ± 0.44, and 2.85 ± 0.69 at 0, 1, 4, 12, 24, and 36 weeks, respectively. The mean GAIS self-reported scores in Group B were 1.77 ± 0.6, 1.92 ± 0.64, 2.08 ± 0.49, 2.46 ± 0.52, 1.77 ± 0.6, 2.38 ± 0.51, and 2.69 ± 0.48 at 0, 1, 4, 12, 24, and 36 weeks, respectively. The mean investigator-rated GAIS scores in Group C were 1.36 ± 0.5, 1.43 ± 0.51, 1.71 ± 0.47, 2.21 ± 0.43, 1.21 ± 0.43, 1.36 ± 0.5, and 1.93 ± 0.27 at 0, 1, 4, 12, 24, and 36 weeks, respectively. The mean GAIS self-reported scores in Group C subjects during follow-up were 1.43 ± 0.51, 1.5 ± 0.65, 1.93 ± 0.47, 2.43 ± 0.65, 1.36 ± 0.5, 1.86 ± 0.36, and 2.21 ± 0.43 at 0, 1, 4, 12, 24, and 36 weeks, respectively.

For the investigator-rated GAIS scores, no significant differences were observed between the three groups at the immediate postinjection time point. At 1 week, significant differences were found between Group A and Group C (*p* = 0.0147). At 4 weeks, significant differences were observed between Group A and Group C (*p* = 0.002), as well as between Group B and Group C (*p* = 0.0394). At 12 weeks, significant differences were found between Group A and both Group B (*p* = 0.0013) and Group C (*p* < 0.0001). No significant differences were observed at the immediate post-second injection (*p* > 0.05). At 24 weeks and 36 weeks, significant differences were observed between Group A and Group C (*p* < 0.0001, *p =* 0010), and between Group B and Group C (*p* = 0.0001, *p* < 0.0001).

**Fig. 7:**
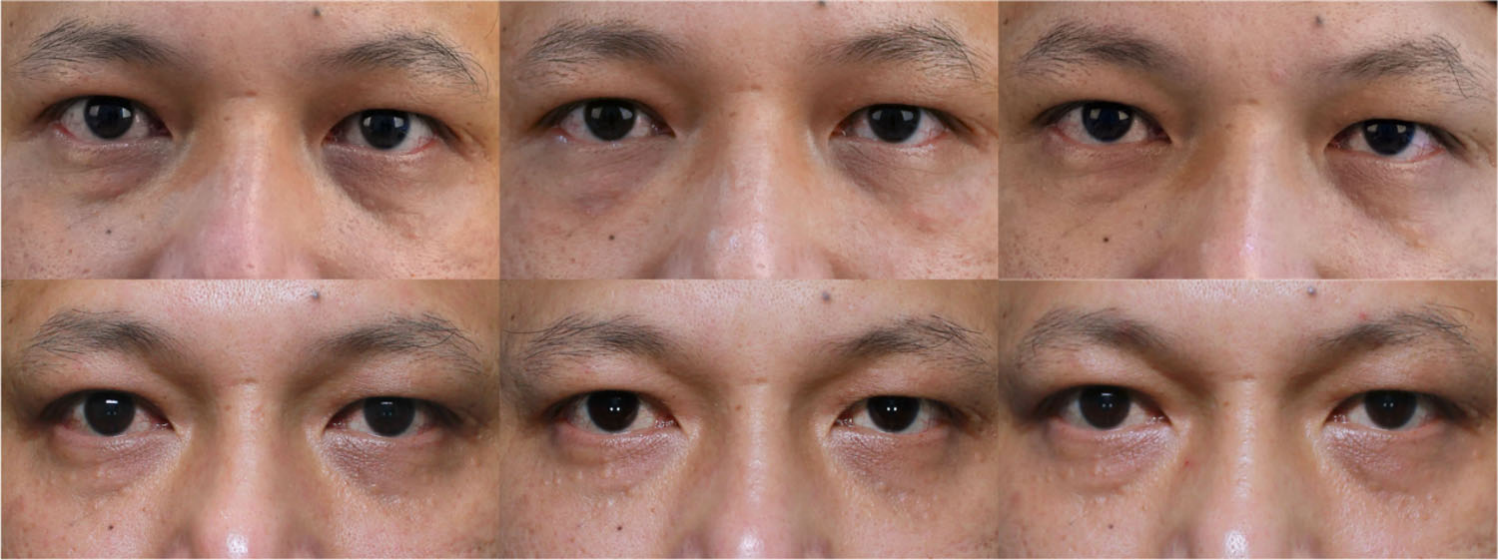
Male, 44 years old, Group A: Collagen injections, Preoperative, immediately postoperation, 4 weeks, 12 weeks, 24 weeks, and 36 weeks

**Fig. 8:**
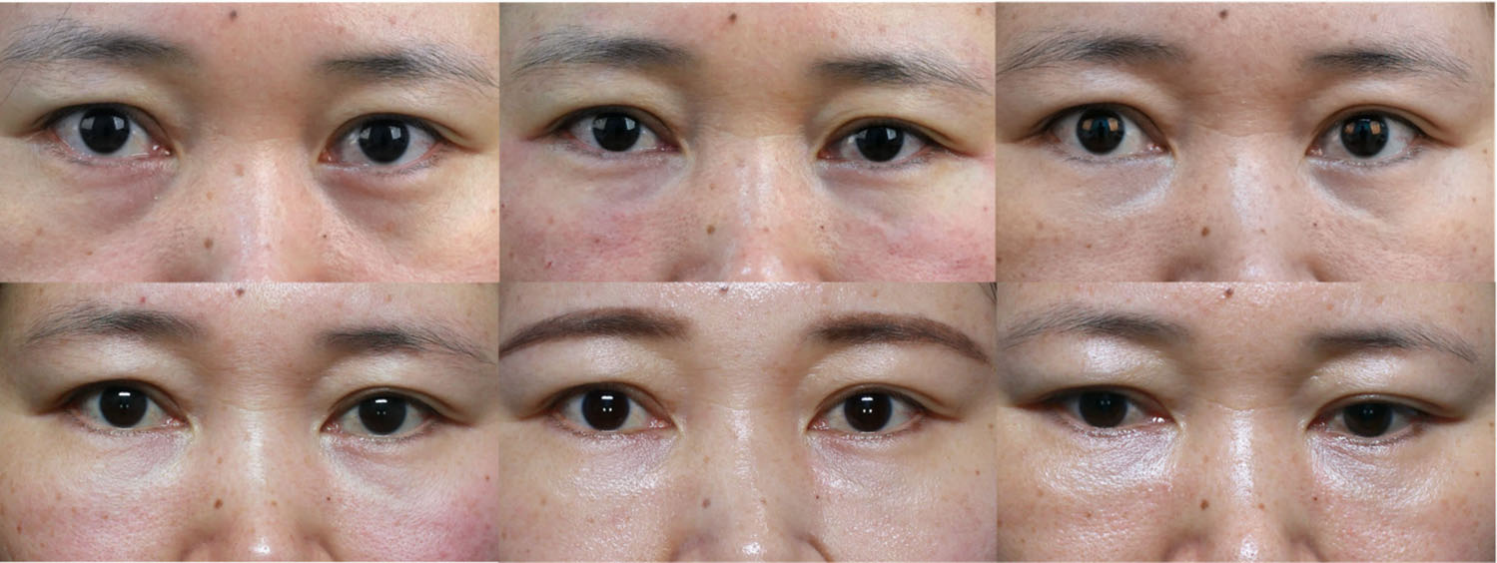
Female, 40 years old, Group B: Hyaluronic acid (Restylane®) injections, Preoperative, immediately postoperation, 4 weeks, 12 weeks, 24 weeks, and 36 weeks

**Fig. 9:**
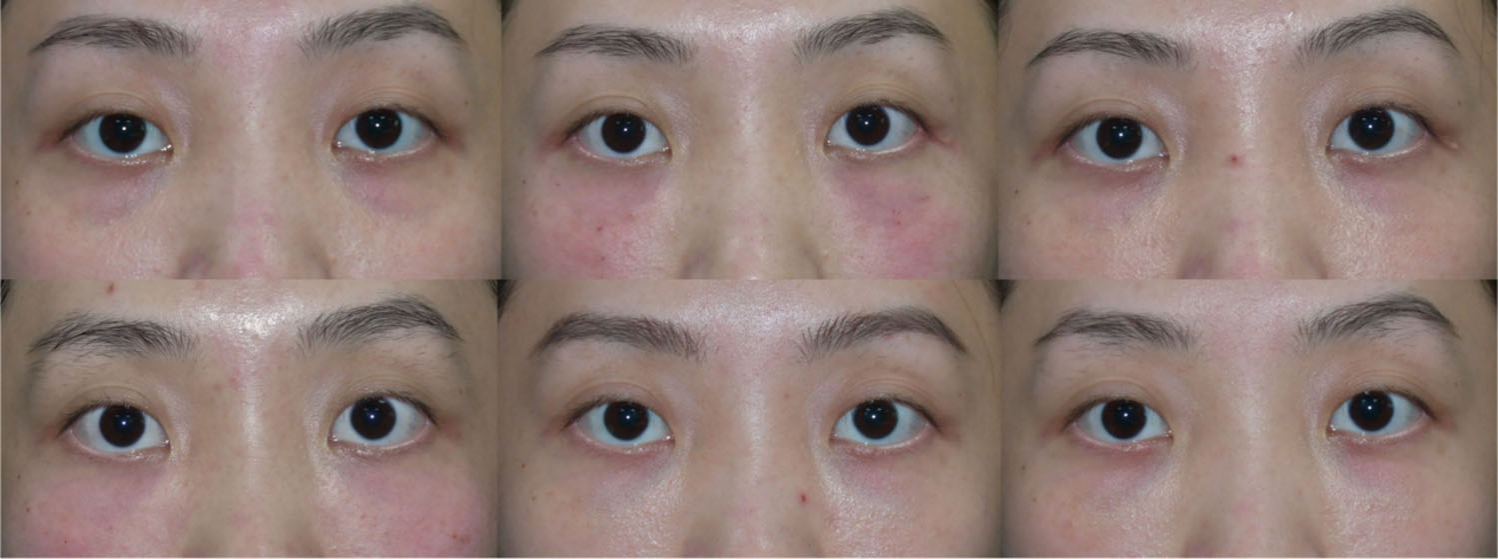
Female, 28 years old, Group C: Combinations of collagen and hyaluronic acid (1:1 ratio) injections, Preoperative, immediately postoperation, 4 weeks, 12 weeks, 24 weeks, and 36 weeks

For the self-reported GAIS scores, no significant differences were found at the immediate postinjection time point. At 1 week, significant differences were observed between Group A and Group C (*p =* 0.0069). At 4 weeks, significant differences were found between Group A and Group C (*p* = 0.0106). At 12 weeks, Group A and Group C showed significant differences (*p* = 0.0409), while no differences were observed at the immediate post-second injection (*p* > 0.05). At 24 weeks, a significant difference was found between Group B and Group C (*p* = 0.0389). At 36 weeks, significant differences were again observed between Group A and Group C (*p* = 0.0052).(Figs. 7, 8 and 9).

## Discussion

Tear trough filler injection is one of the most challenging facial rejuvenation procedures because of the multifactorial nature of periorbital aging, which involves changes in bone, ligament, muscle, fat, and skin. Each patient's tear trough deformity exhibits unique clinical characteristics, requiring a thorough evaluation and a personalized treatment approach [11]. This study aimed to assess the efficacy of a combination of collagen and low-molecular-weight HA for treating tear trough deformities. Our results indicated that this combination approach significantly improves tear trough deformities, particularly in terms of volume restoration and long-lasting effects.

In our study, the improvement rate of the collagen group alone tended to decrease from one month onward. After the second supplementary treatment at 3 months, the improvement rate gradually increased and stabilized, indicating that supplementary injection is necessary when collagen alone is injected into the lacrimal groove. The group treated with hyaluronic acid alone maintained a good improvement rate at the 3-month follow-up; however, this group had a higher risk of the Tyndall effect. Group C, which received a combination treatment, exhibited good results in terms of the improvement rate and adverse events. After two treatments, the combination treatment showed superior results compared with either HA or collagen alone at 9 months post-baseline injection. While no significant differences were observed at 1 month, the 3-month follow-up assessment revealed that the combination group exhibited greater sustained improvements in both tear trough rating scales and global aesthetic improvement scores. The rationale for this dual-layer approach is to leverage collagen’s immediate volumizing effect in the superficial layers to address skin textural irregularities, whereas HA’s deeper volumizing properties provide structural support to counteract ligamentous laxity and restore facial contours. In noninferiority testing, HA monotherapy (Group B) was used as the positive control, and the combination treatment (Group C) was shown to have comparable efficacy, with a slight advantage in durability over the monotherapy groups.

In line with our findings, recent studies, such as Liu et al. (2024), have also explored the combination of collagen and hyaluronic acid (HA) for tear trough deformities [12]. Their study found that this combination not only reduced the Tyndall phenomenon but also provided more consistent and durable improvements compared to HA alone. This supports our results, where Group C (collagen + HA) exhibited superior improvement at 36 weeks, reflecting the benefits of combining HA's long-lasting effects with collagen's skin-enhancing properties. Moreover, Liu et al. (2024) emphasized the combination's ability to address both deep volumization and superficial texture, making it a more effective option for complex periocular aging. Our study builds on these findings, highlighting that the combination of collagen and HA reduces the Tyndall effect—commonly associated with superficial HA injections—while offering both immediate and long-term volume restoration. Collagen targets superficial skin issues, while HA provides deeper structural support, resulting in a more natural and durable outcome than either filler used alone [13].

The effectiveness of the combination treatment is likely due to the synergistic effects of collagen and HA. When collagen (types I/III) is injected into the superficial layers, it helps to restore skin structure and texture, while HA targets deeper volume loss and addresses the structural deficits that contribute to tear trough deformities. Additionally, the myomodulatory effect of the injected fillers may also contribute to improvement by providing support to the orbicularis oculi muscle, which plays a role in the tear trough’s appearance. This dual-layer approach effectively minimizes the risk of complications such as the Tyndall effect while improving the overall aesthetic outcome.

In comparison, collagen injections alone offer certain advantages but also present limitations. One key advantage of collagen is its ability to improve skin texture and tone by restoring structure to the dermis, which is crucial in addressing superficial skin laxity and fine wrinkles. However, one of the main disadvantages of using collagen alone is its relatively short duration of effect. Collagen-based fillers typically last 1–2 months, after which the results begin to diminish. Compared with HA, which tends to last longer in deeper layers, this shorter duration means that patients may require more frequent treatments to maintain the desired outcome. In our study, while collagen alone was effective at the 1-month follow-up assessment, it did not yield sustained improvements at the 3-month follow-up assessment like the combination treatment. This shorter retention time is a significant limitation when collagen monotherapy is applied for tear trough deformities.

In terms of safety, this study carefully monitored adverse events, including swelling, bruising, and the Tyndall effect. Minor complications, such as swelling and bruising, were the most commonly observed adverse events and typically resolved within 1–2 weeks without affecting the overall results. Importantly, the occurrence of the Tyndall effect was notably lower in the combination treatment group than in the HA monotherapy group, further emphasizing the benefit of using a mixture of collagen and HA for tear trough rejuvenation. No serious adverse events were reported, and the safety profile of the combination treatment was manageable and clinically acceptable.

Despite these promising results, this study has several limitations. First, the sample size was relatively small, although the research was conducted across multiple centers. While this increases the diversity of participants, the sample size still limits the statistical power and generalizability of the findings. Second, the follow-up period was limited to 9 months. While the study provides valuable insights into the short- and medium-term effects of the combination treatment, a longer follow-up period, such as 12 months or more, would likely provide more clarity regarding the long-term durability of the treatment. A longer follow-up would also help assess whether the distinction between the combination treatment and HA or collagen alone becomes more apparent over time.

Our results showed that the combination treatment (Group C) provided sustained improvements compared to both hyaluronic acid and collagen treatments, with a plateau occurring only at the 36-week follow-up, suggesting the importance of continued treatment to maintain results.

In future research, extending the follow-up period and increasing the sample size across multiple centers would strengthen the external validity of the findings. Moreover, investigating the potential benefits of combining HA and collagen with other fillers, such as calcium hydroxyapatite or poly-L-lactic acid, could further enhance treatment effectiveness and prolong the duration of the effects. Exploring different combinations or modifications of injectable materials may lead to more personalized and effective approaches for tear trough rejuvenation.

Overall, the results of this study demonstrate that the combination of collagen and hyaluronic acid (HA) is effective and safe for correcting moderate periocular aging in Chinese subjects over 18 years of age.

## Conclusion

The combination of collagen and hyaluronic acid (HA) for tear trough rejuvenation is a promising approach. Our study revealed that this dual-layer technique provides longer-lasting results while minimizing complications such as the Tyndall effect. While there are several limitations, the results support the potential clinical use of this combination therapy for tear trough rejuvenation. Further studies with larger sample sizes and longer follow-up periods will provide a more comprehensive understanding of the benefits and long-term outcomes of this combination treatment.

## Supplementary Information

Below is the link to the electronic supplementary material.

Supplementary file1 (MP4 5633 kb)
